# Continuous Blood Pressure Estimation From Electrocardiogram and Photoplethysmogram During Arrhythmias

**DOI:** 10.3389/fphys.2020.575407

**Published:** 2020-09-09

**Authors:** ZengDing Liu, Bin Zhou, Ye Li, Min Tang, Fen Miao

**Affiliations:** ^1^Chinese Academy of Sciences Key Laboratory for Health Informatics, Shenzhen Institutes of Advanced Technology, Shenzhen, China; ^2^Joint Engineering Research Center for Health Big Data Intelligent Analysis Technology, Shenzhen Institutes of Advanced Technology, Chinese Academy of Sciences, Shenzhen, China; ^3^State Key Lab of Cardiovascular Disease, Fuwai Hospital, National Center for Cardiovascular Diseases, Chinese Academy of Medical Sciences and Peking Union Medical College, Beijing, China

**Keywords:** arrhythmias, continuous blood pressure, electrocardiogram, photoplethysmogram, machine learning algorithms

## Abstract

**Objective:**

Continuous blood pressure (BP) provides valuable information for the disease management of patients with arrhythmias. The traditional intra-arterial method is too invasive for routine healthcare settings, whereas cuff-based devices are inferior in reliability and comfortable for long-term BP monitoring during arrhythmias. The study aimed to investigate an indirect method for continuous and cuff-less BP estimation based on electrocardiogram (ECG) and photoplethysmogram (PPG) signals during arrhythmias and to test its reliability for the determination of BP using invasive BP (IBP) as reference.

**Methods:**

Thirty-five clinically stable patients (15 with ventricular arrhythmias and 20 with supraventricular arrhythmias) who had undergone radiofrequency ablation were enrolled in this study. Their ECG, PPG, and femoral arterial IBP signals were simultaneously recorded with a multi-parameter monitoring system. Fifteen features that have the potential ability in indicating beat-to-beat BP changes during arrhythmias were extracted from the ECG and PPG signals. Four machine learning algorithms, decision tree regression (DTR), support vector machine regression (SVR), adaptive boosting regression (AdaboostR), and random forest regression (RFR), were then implemented to develop the BP models.

**Results:**

The results showed that the mean value ± standard deviation of root mean square error for the estimated systolic BP (SBP), diastolic BP (DBP) with the RFR model against the reference in all patients were 5.87 ± 3.13 and 3.52 ± 1.38 mmHg, respectively, which achieved the best performance among all the models. Furthermore, the mean error ± standard deviation of error between the estimated SBP and DBP with the RFR model against the reference in all patients were −0.04 ± 6.11 and 0.11 ± 3.62 mmHg, respectively, which complied with the Association for the Advancement of Medical Instrumentation and the British Hypertension Society (Grade A) standards.

**Conclusion:**

The results indicated that the utilization of ECG and PPG signals has the potential to enable cuff-less and continuous BP estimation in an indirect way for patients with arrhythmias.

## Introduction

Heart arrhythmia, also known as irregular heartbeat or cardiac dysrhythmia, is a group of conditions characterized by a heartbeat that is irregular, too slow, or too fast (Kligfield et al., 2007). Arrhythmias can be classified into two types according to their origin: ventricular and supraventricular arrhythmias (Jenkins et al., 1979). Arrhythmias is an age-related disease (Hatch et al., 2011; Khurshid et al., 2018). For instance, the incidence of paroxysmal supraventricular arrhythmias is 14.8 per 100,000 person-years in adults aged 18 to 24 years, but in persons aged ≥65 years, the rate is increased to 231.9 per 100,000 person-years (Go et al., 2018). It is estimated that by 2050, supraventricular-related arrhythmias will affect approximately nine million individuals aged >60 years in China (Tse et al., 2013). Therefore, with the growth of the aging population, arrhythmias will bring an increasing burden and challenge to public healthcare management.

Blood pressure (BP) is an important physiological parameter of the human body. BP is closely related to the occurrence, development, and prognosis of arrhythmias (Yildirir et al., 2002). Continuous (beat to beat) BP monitoring can provide detailed BP changes to facilitate BP management (Escobar-Restrepo et al., 2018). Thus, continuous BP monitoring is of importance for the health management of patients with arrhythmias. However, because of the irregular heart rate and stroke volume during arrhythmias, considerable variation (beat to beat BP changes) occurs in continuous BP. Regular intermittent BP measurement (e.g., auscultatory technique) was demonstrated to with high bias in such situations (Cohen and Townsend, 2017). In the clinical setting, the intra-arterial method is recommended for the BP measurement of patients with arrhythmias (Alpert et al., 2006). Nevertheless, the intra-arterial approach involves the catheter inserted into an arterial by physicians and is thus too invasive in routine application. A non-invasive approach that can provide continuous BP measurement is thus of great significance for the routine healthcare for patients with arrhythmias.

The existing non-invasive approaches, including artery applanation tonometry and volume clamp technologies, have been used for continuous BP measurement, but they are complicated and uncomfortable for long-term monitoring (Peter et al., 2014). Moreover, the accuracy of these approaches is low, particularly in patients with arrhythmias (Kim et al., 2014; Ilies et al., 2015). Cuff-less methods, which can provide unobtrusive continuous and long-term BP monitoring, have received increasing attention in recent years. Typical cuff-less methods include pulse transit time (PTT)-based and multi-parameter–based approaches. PTT-based approaches have been widely studied, and they are the most popular methods for continuous BP measurement (Mukkamala et al., 2015; Huynh et al., 2018; Liu J. et al., 2018; Yang and Tavassolian, 2018; Ding and Zhang, 2019). As a potential BP measurement indicator, PTT refers to the time taken for a pulse wave to propagate between two locations in the cardiovascular system. It can be calculated from two pulse signals generated by the cardiovascular system, such as electrocardiogram (ECG) and photoplethysmogram (PPG) signals, or two peripheral PPG signals. A novel approach of measuring arteriolar PTT based on multi-wavelength PPG was proposed recently by Liu J. et al. (2018); they provided compact and inexpensive wearable healthcare electronics for continuous BP measurement. However, because of the fixed hypothesis of the BP–PTT relationship, PTT-based approaches have low accuracy and robustness because other indicators such as vascular tone, physiological statue, and individual affect the BP–PTT relationship. To improve the accuracy of PTT-based approaches, potential BP variation indicators, such as time-, slope-, ratio-, and area-based features, were extracted from ECG and PPG signals and considered along with PTT to construct a multi-parameter–based model for BP estimation (Baek et al., 2009; Ding X.-R. et al., 2016; Kachuee et al., 2017; Miao et al., 2017; Lin et al., 2018; Liu Z.-D. et al., 2018). For instance, Ding X.-R. et al. (2016) proposed a new indicator, namely the PPG intensity ratio (PIR), which can reflect arterial diameter changes and thus indicate BP variation. Their experimental results demonstrated that the model using a combination of the PTT and PIR had better accuracy in tracking BP than that did the model based on PTT alone. Lin et al. (2018) also proposed additional PPG indicators for improving the performance of PTT-methods for continuous BP measurement. Kachuee et al. (2017) extracted physiological parameters and whole-based features from ECG and PPG signals and then established a continuous BP estimation model based on machine learning algorithms.

PTT-based and multi-parameter–based models enable cuff-less continuous BP measurement based on ECG and PPG signals. However, most of them have been mainly applied in individuals with regular heartbeat; a few studies using intermittent or Finapres BP as the gold reference have demonstrated attenuated performance for these models in patients with cardiovascular diseases (Wagner et al., 2010; Liu et al., 2014; Ding X. et al., 2016). Studies on cuff-less and continuous BP measurement for patients with arrhythmias are limited. Continuous BP is with a high degree of fluctuations under arrhythmias, and these fluctuations can be attributed to vessel compliance, unstable cardiac contractility, and consequent blood volume changes. By investigating the indicators causing BP variation from physiological signals (such as ECG and PPG signals) during arrhythmias and using these indicators to develop the model for BP estimation, continuous BP estimation in a cuff-less way can be realized for patients with arrhythmias.

The main purpose of this study is to investigate the feasibility of using ECG and PPG signals for continuous (beat-to-beat) systolic BP (SBP) and diastolic BP (DBP) estimation during arrhythmias based on machine learning algorithms. Potential indicators extracted from ECG and PPG signals for large BP variation during arrhythmias were studied and then used to develop the BP model. The performance was validated using a clinical experiment involving 35 patients with arrhythmias, with the invasive technique as the gold reference. Since the hemodynamic responses to changes in BP vary considerably among individuals, as well as the difficulty of collecting too much data under arrhythmias, personalized modeling was used in this study. The potential clinical application of this work is that after initial BP calibration, it may provide accurate long-term BP tracking for patients with arrhythmias in a non-invasive way.

## Materials and Methods

### Experimental Protocol

#### Study Population

A total of 40 clinically stable patients with arrhythmias who required radiofrequency catheter ablation through the femoral artery at FuWai Hospital, Chinese Academy of Medical Sciences were evaluated observing the inclusion and exclusion criteria. Exclusion criteria included patients diagnosed with (1) malignant tumors; (2) severe organic heart diseases (myocardial infarction, congenital heart disease, severe valvular disease, and severe atrioventricular block); (3) arterial stenosis (upper extremity artery, thoracoabdominal aortic stenosis, and hip artery stenosis). Five patients were excluded because they met the exclusion criteria: one patient had malignant tumor; three patients had severe organic heart diseases; 1patient had arterial stenosis. Flow chart of study population is shown in Supplementary Figure S1. This study was approved by the institutional ethics review board of Fuwai hospital (Approval No. 2019-1239). All enrolled patients signed informed consent forms before the study. The protocol was registered on www.chictr.org.cn (registration number: ChiCTR2000031170).

#### Signal Acquisition and Pre-processing

**FIGURE 1:**
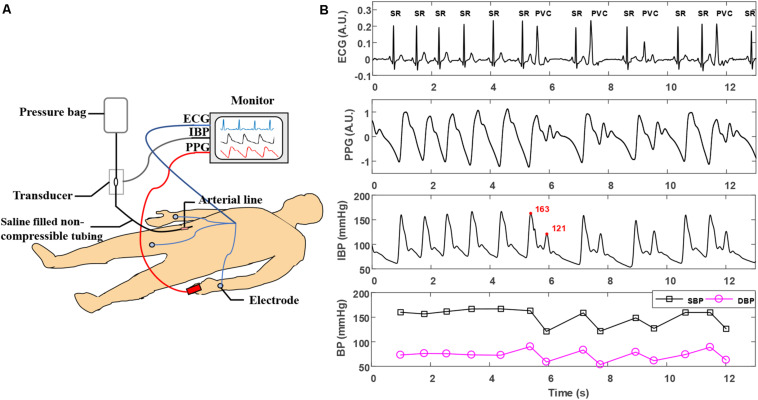
**(A)** Experimental scene of simultaneous acquisition of ECG, PPG, and IBP signals. **(B)** Typical ECG, PPG, and IBP waveforms recorded in arrhythmias. The type of each beat is labeled in the ECG waveforms. The two adjacent beats in the IBP waveforms with the largest changes in SBP are marked in the red font. ECG, electrocardiogram; IBP, invasive blood pressure; PVC, premature ventricular contraction; PPG, photoplethysmogram; SR, sinus rhythm; SBP, systolic blood pressure.

Before the operation, a multi-parameter monitoring system (BeneVision N12, Shenzhen Mindray Bio-Medical Electronics, China) was used to acquire synchronous ECG, PPG, and invasive BP (IBP) signals for each patient in the supine position. The sampling rate for ECG, PPG, and IBP collection was set to 250 Hz. ECG and PPG signals were acquired using I-lead ECG electrodes placed on the left and right arms and the right leg, and a PPG sensor was placed on the left index finger. For IBP monitoring, an arterial catheter was inserted into the right femoral artery and then connected to the N12 monitor. Calibration to atmospheric zero was performed before initiation of each recording by opening the pressure transducer of the catheter to atmospheric pressure. ECG, PPG, and IBP waveform recordings were taken for at least 10 min. All procedures were performed by experienced professionals in the standard ablation operating room of FuWai Hospital. The experimental conditions are illustrated in Figure 1A.

Reference SBP and DBP values were defined as the maximum and minimum values of IBP waveforms in each cardiac cycle. For each patient, a poor PPG signal quality is defined as the deviation of the energy of the PPG signal from the average energy by more than twice the standard deviation. The method for calculating the energy of PPG signal was described in details in previous research (Lin et al., 2019).

### Feature Extraction

**FIGURE 2:**
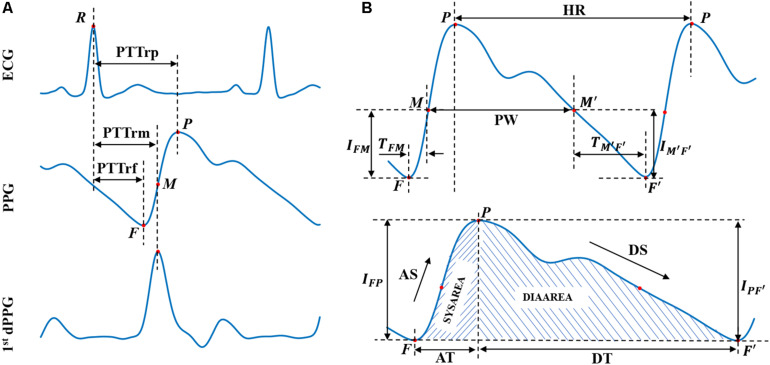
Extracted physiological features from ECG, PPG, and 1st dPPG in a cardiac cycle. First dPPG indicates the first derivative of PPG. **(A)** PTT values extraction. **(B)** PPG features extraction. *R* represent the R-wave peak of ECG; *F*/*F*′ and *P* represent the foot and peak of the PPG waveform, respectively, and letter *M* (and its corresponding point *M*′) represents the peak of the first derivative of PPG. Abbreviations and detailed definitions show in Table 1.

Based on the physiological background between BP and corresponding ECG and PPG signals, 15 crucial features (numbered from 1 to 15 and listed in Table 1) containing cardiovascular information were extracted from ECG and PPG signals in each cardiac cycle for BP estimation. Figure 2 illustrates the extracted features, and Table 1 summarizes the detailed description and calculation methods for the extracted features. The extracted features are described as follows: according to different distal timing reference points selected in PPG signals, three PTTs (i.e., the time interval from the R wave peak of ECG to the foot of PPG, the peak of the first derivative of PPG, and the peak of PPG, respectively) were calculated from ECG and PPG signals in the same cardiac cycle (Miao et al., 2017, 2020). The corresponding PTTs are called PTTrf, PTTrm, and PTTrp, respectively (see Figure 2A). Eight PPG features (Features 4 to 11), including time-, slope- and area-based features (Lin et al., 2018), were then calculated from the beat-to-beat ascending (Features 4 to 7) and descending edges (Features 8 to 11) of the PPG waveform. Since PIR, pulse width, and heart rate were previously reported as effective indicators for BP estimation (Baek et al., 2009; Arza et al., 2013; Ding X.-R. et al., 2016), they were also calculated in the study. Besides, another indicator K value (Feature 15) from the whole informative representation of the beat-to-beat PPG waveform was found to be a potential feature for BP estimation in our previous study (Miao et al., 2020); it was also included in this study. Therefore, a total of 15 features were calculated for BP estimation during arrhythmias. Note that the feature extraction depends on the precise characteristic points location on the PPG signals. However, due to the occurrence of irregular and inappropriate PPG shapes in patients with cardiovascular diseases, it may be difficult to accurately locate the position of characteristic points in their PPG waveforms and then to extract the PPG features (e.g., dicrotic wave related features) (Somayyeh et al., 2019). In this study, the extracted PPG features (Features 4 to 15, listed in Table 1) only rely on the location of three easily detectable points (i.e., the foot and peak of the PPG waveform and the peak of the first derivative of PPG), which improve the robustness of the features extracted from each cardiac cycle.

**TABLE 1:** Definitions and calculation methods of the extracted features.

Features	Calculations	Definitions
(1) Pulse transit time R-foot	PTTrf	Time delay from the R-peak of ECG to the foot of PPG
(2) Pulse transit time R-middle	PTTrm	Time delay from the R-peak of ECG to the peak of the first derivative of PPG
(3) Pulse transit time R-peak	PTTrp	Time delay from the R-peak of ECG to the peak of PPG
(4) Ascending time	AT	Time span between the foot and peak of PPG in ascending edge
(5) Ascending slope	AS = I_*FP*_/AT	Ratio of PPG peak-foot point intensity difference in ascending edge (*I*_*FP*_) to ascending time
(6) first part of AS	FAS = *I*_*FM*_/*T*_*FM*_	Ratio of PPG *M* point intensity (*I*_*FM*_) to the duration from the foot point to *M* point of PPG (*T*_*FM*_)
(7) Ascending area	$\text{SYSAREA}=\sum_{i=F}^{P}\left(I_{i}-I_{F}\right)$	Area under the ascending portion of the PPG waveform
(8) Descending time	DT	Time span between the peak and foot of the PPG in Descending edge
(9) Descending slope	DS = *I*_*PF*′_/*DT*	Ratio of PPG peak-foot point intensity difference in descending edge (*I*_*PF’*_) to descending time
(10) First part of DS	FDS = *I*_*M*′*F*′_/*T*_*M*′*F*′_	Ratio of PPG *M*′ point intensity (*I*_*M’F’*_) to the duration from the *M*′ to foot point of PPG (*T*_*M’F’*_)
(11) Descending area	$\text{DIAAREA}=\sum_{i=p}^{F^{\prime }}\left(I_{i}-I_{F^{\prime }}\right)$	Area under the descending portion of the PPG waveform
(12) PPG intensity ratio	PIR (Ding X.-R. et al., 2016)	Ratio of PPG peak intensity to foot intensity
(13) Pulse width	PW	Time span between the *M* point and *M*′point of PPG
(14) Heart rate	HR	Time span between two adjacent peaks of PPG
(15) *K* value	K (Miao et al., 2020)	PPG characteristic value

*Point *M* (and its corresponding point *M*′ in descending edge) represents the maximum derivative point of PPG; $\sum_{i=F}^{P}\left(I_{i}-I_{F}\right)$ represents the cumulative sum of intensity difference of PPG from point foot (*F*) to point peak (*P*) in ascending edge; $\sum_{i=P}^{F^{\prime }}\left(I_{i}-I_{F^{\prime }}\right)$ presents the cumulative sum of intensity difference of PPG from point peak (*P*) to point foot (*F*′) in descending edge. PPG, photoplethysmogram.*

### Model Construction and Validation

Four machine learning algorithms were implemented to establish the relationship between the extracted features and the reference BP values by using the Scikit-learn library in a Python programming environment (Pedregosa et al., 2011). It is important to note that the proposed method estimates the BP in terms of beat to beat, with the estimation frequency in seconds (depending on the patient’s heart rate), which can also be regarded as continuous (Kachuee et al., 2017).

(1) Decision Tree Regression (DTR): decision trees are supervised machine learning models for predicting a target in the form of a tree structure, which consists of many nodes and branches (Quinlan, 1986). When the target variable of a decision tree is continuous, the model is called DTR. In this study, a DTR model was trained using a built-in function (Decision Tree Regressor) with default parameters from the Scikit-learn library.
(2) Support vector machine regression (SVR): support vector machines also belong to supervised learning algorithms. They can efficiently perform classification or solve a regression problem by non-linearly mapping input feature vectors into higher dimensional spaces by using a kernel function, such as radial basis function (RBF), polynomial function, and Sigmoid function (Awad and Khanna, 2015). In this study, an SVR function with RBF kernel in the Scikit-learn library was used for training and testing the BP estimation model. The penalty parameter and kernel parameter for each patient were selected through an exhaustive grid search (Lee and Lin, 2000).
(3) Adaptive Boosting Regression (AdaboostR): AdaboostR, in contrast to the SVR, is an ensemble learning algorithm that creates a strong estimator from many weak estimators (e.g., decision trees). In AdaboostR, the predictions of weak estimators are combined into a weighted sum that represents the final prediction (Freund et al., 1999). Compared with other complex and strong models, AdaboostR models are less prone to overfitting (Kachuee et al., 2017). For training the regression model, an AdaboostR function (AdaBoostRegressor) with default parameters in the Scikit-learn library was used.
(4) Random forest regression (RFR): Random forests are another popular ensemble method, where the final prediction is the composite outcome from many weak estimators (Breiman, 2001). Compared with the AdaboostR, the base estimators in the random forest are trained independently, so the random forest models require less training process (Liaw and Wiener, 2002). Moreover, random forests have the advantage of measuring the importance of variables (Cutler et al., 2012). Here, an RFR function (RandomForestRegressor) with 50 trees and other default parameters in the Scikit-learn library (Pedregosa et al., 2011) was used for model training. Then the feature scores returned by the trained model were used to evaluate the importance of the extracted features.

The machine learning algorithms mentioned above were implemented to construct an individual BP estimation model for each patient. The difference between the estimated BP (SBP or DBP) with the proposed regression algorithm and the reference BP (invasive SBP or DBP) is defined as the estimation error. Four metrics, including root-mean-square error (RMSE), mean error (ME), standard deviation of error (STD), and mean absolute error (MAE) between the predicated and the reference BP values were calculated to evaluate the estimation accuracy of different algorithms. The correlation coefficient (*r*-value) was also included as a metric to measure the consistency between the predicated BP values and the references (invasive SBP/DBP values). Formulas for these metrics are presented in Supplementary Text S1.

**FIGURE 3:**
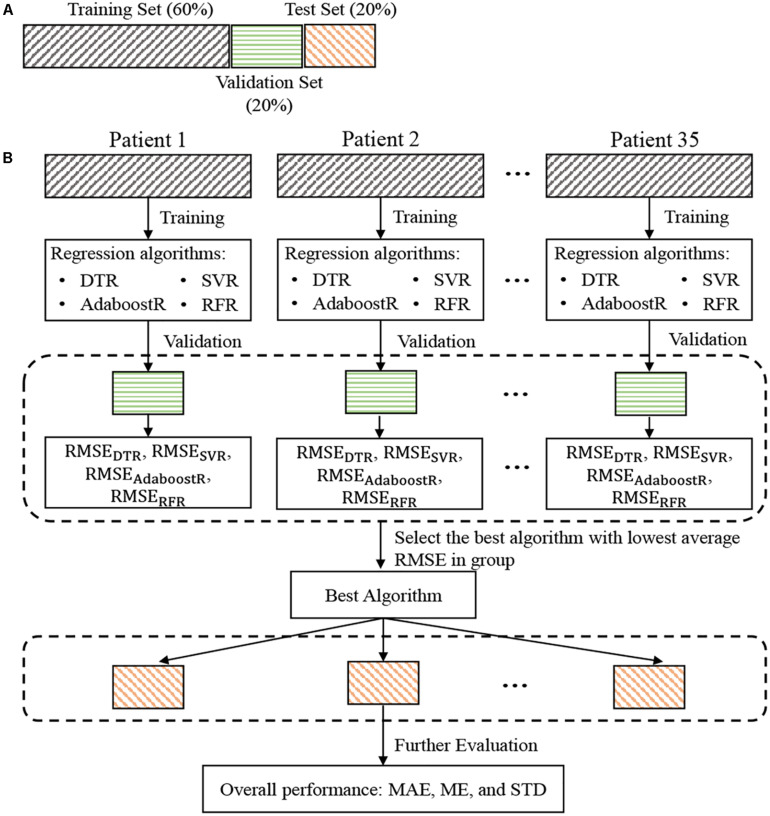
Dataset partition for each patient **(A)** and flowchart of blood pressure estimation model construction and evaluation **(B)**. RMSE_DTR_, RMSE_SVR_, RMSE_AdaboostR_, and RMSE_RFR_ represents the root mean square error between the reference and the estimated blood pressure values by the DTR, SVR, AdaboostR, and RFR, respectively. RMSE, root-mean-square error; DTR, decision tree regression; SVR, support vector machine regression; AdaboostR, adaptive boosting regression; RFR, random forest regression.

Details of the dataset partition and the construction and evaluation process for the individual model were illustrated in Figure 3. For each patient, the data were divided into training, validation, and test set, with a ratio of 6:2:2. Train the regression algorithms on the training set and then validate them on the validation set to select the best model. More specifically, the RMSE was used to evaluate the estimation performance of each trained regression algorithm on the validation set for each patient. The trained regression algorithm with the lowest average (mean value ± standard deviation) RMSEs in all patients was considered as the best algorithm. The performance of this best algorithm in all patients was then further evaluated in terms of the values of ME ± STD and MAE according to the two most applied BP devices evaluation standards, the Association for the Advancement of Medical Instrumentation (AAMI) and British Hypertension Society (BHS). Statistical significance of the differences between the performance of different algorithms was also assessed using Student’s *t*-test. A *p*-value <0.05 considered as statistically significant.

### Feature Importance Assessment

Identifying features that are critical for BP estimation under arrhythmias is of great clinical significance. Moreover, knowing which features in a model are important for its predicting results is valuable for selecting features, reducing data dimensionality, and improving the operability the of model. To evaluate the importance of each feature during arrhythmias in the study population, a relative weight-based strategy was proposed. The important relative level of feature *f*_*j*_ in patient *p*_*i*_ can be expressed as

(1) $$F\,I_{i,j}=\omega_{i,j}/\sum_{j=1}^{M}\omega_{i,j}$$

where ω_*i,j*_, with *i* = 1,…,*N* and *j* = 1,…,*M*, is the weight score of feature *f*_*j*_ returned by the RFR model in patient *p*_*i*_, and *N* and *M* are the total number of patients and features in the experiment, respectively.

The important level of feature *f*_*j*_ in the population was then calculated by averaging the feature level *FI*_*i,j*_:

(2) $$F\,I=\sum_{i}^{N}F\,I_{i,j}/N$$

## Results

### Baseline Characteristics and of the Selected Study Population

Based on inclusion and exclusion criteria, 35 clinically stable patients with arrhythmias who required radiofrequency catheter ablation through the femoral artery (age: 43.87 ± 14.01 years; 20 were men) were included in the analysis. Among the 35 patients, 15 had ventricular arrhythmias, and 20 had supraventricular arrhythmias.

### Acquired Signals of ECG, PPG, and IBP

After removing the PPG signal segments with poor signal quality, a final of 17,796 beats of ECG, PPG, and IBP signals were obtained from 35 patients. The number of beats for each type (manually marked by two cardiologists) and beat-to-beat SBP/DBP change for each type were summarized in Table 2. Noted that the BP change for each beat (sinus beat or non-sinus beat) is defined as the difference between the BP of the beat and the BP of its nearest sinus beat. Figure 1B presents a typical example of synchronous ECG, PPG, and IBP waveforms and continuous reference SBP and DBP measurements collected from a patient with arrhythmia. Figure 1B illustrates that during arrhythmias, irregularity in ECG signals causes large IBP and beat-to-beat SBP and DBP variations, accompanied by irregular patterns in PPG signals, such as peak-to-peak interval (i.e., the time interval between two adjacent peaks of PPG) and PPG amplitude. With the occurrence of ventricular premature beats in ECG, the maximum SBP variation is >40 mmHg (as shown in IBP waveforms in Figure 1B), suggesting a large variation in continuous BP during arrhythmias.

**TABLE 2:** Number of beats and beat-to-beat BP changes (mean ± standard derivation) of different types of beats.

Type of beat	Beats	BP Changes^a^ (mmHg)	
		SBP	DBP
Sinus rhythm	6 212	2.13 ± 3.06	1.55 ± 3.89
Ventricular arrhythmias	3 367	33.85 ± 26.03	11.06 ± 6.95
Supraventricular arrhythmias	8 217	15.30 ± 8.51	5.72 ± 4.72
Overall	17 796	9.93 ± 14.61	3.96 ± 5.58

*^*a*^BP change for each beat is defined as the difference between the BP of the beat and the BP of its nearest sinus beat. BP, blood pressure; DBP, diastolic blood pressure; SBP, systolic blood pressure.*

### Machine Learning Algorithm Selection

**FIGURE 4:**
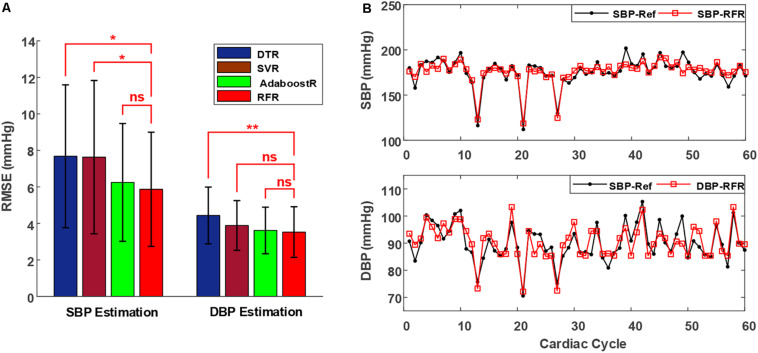
**(A)** Performance (mean value ± standard deviation of RMSEs) of different regression algorithms to estimate SBP and DBP in the validation set of all patients. Triple asterisks “*,” “**,” and “ns” indicate statistical significance at *p* < 0.05, *p* < 0.01, and *p* > 0.05, respectively. **(B)** Estimated beat-to-beat SBP and DBP comparisons in a representative patient, with the proposed method indicated in red and the reference shown in black. Abbreviations show in Figure 3.

Figure 4A illustrates the average group RMSEs of various regression algorithms on the validation set of all patients. The results were based on the mean value ± standard deviation of RMSEs. The RFR model afforded significantly smaller RMSEs in SBP estimation (5.87 ± 3.13 mmHg) than did the DTR (7.68 ± 3.91 mmHg) and SVR (7.63 ± 4.12 mmHg) models; however, although the SBP estimation RMSEs of the RFR model were also smaller than those of the AdaboostR model (6.24 ± 3.22 mmHg), the difference was non-significant. Moreover, the RMSEs of the RFR model in DBP estimation were 3.52 ± 1.38 mmHg, which were comparable to that of the SVR (3.88 ± 1.35 mmHg) and AdaboostR (3.62 ± 1.28 mmHg) models, but significantly smaller than that of the DTR model (4.44 ± 1.56 mmHg). Therefore, based on its higher performance (i.e., lower RMSEs) in BP estimation and faster training capacity compared with the AdaboostR model (Liaw and Wiener, 2002), the RFR model was selected as the best estimator algorithm.

Figure 4B presents a typical example of a patient’s beat-to-beat comparison between the reference BP values (SBP-Ref and DBP-Ref, marked in black) and estimated BP values (SBP-RFR and DBP-RFR, marked in red) by using the RFR model. In the reference BP values, the maximum variations in SBP and DBP are higher than 70 and 20 mmHg, respectively, as shown in the 21-*th* and 22-*th* cycles in Figure 4B. The estimated BP values with the proposed approach could follow these large BP variations accurately. Therefore, the proposed method shows a good performance in BP measurement for patients with arrhythmias.

### Evaluation Based on the Association for the Advancement of Medical Instrumentation and British Hypertension Society Standards

The overall performance of the proposed method (the best model based on RFR algorithm) was further evaluated against the AAMI (Association for the Advancement of Medical Instrumentation [AAMI], 2009) and BHS (O’Brien et al., 1990) standards. The AAMI standard require the ME and STD of BP measurement devices to be ≤5 and ≤8 mmHg, respectively, whereas based on the BHS standard, BP measurement devices are graded based on their cumulative percentage (CP) of MAE under the three thresholds of 5, 10, and 15 mmHg.

Table 3 presents the performance of the proposed method in estimating BP by using the RFR algorithm in all patients, in which the performance was calculated based on the estimation errors in the test set of all patients. Details of the estimation performance of the proposed method under different types of beats (as categorized in Table 2) were summarized in Supplementary Table S1. Overall, ME ± STD values for the SBP and DBP estimation were −0.04 ± 6.11 and 0.11 ± 3.62 mmHg, respectively, indicating that the proposed method for the SBP and DBP estimation meets the AAMI standard for patients with arrhythmias in the present scenarios. According to the BHS standard, the proposed method is consistent with grade A for both SBP and DBP estimation. Besides, according to the AAMI standard (Association for the Advancement of Medical Instrumentation [AAMI], 2009), an evaluation population of at least 35 individuals is required when the BP measurement devices are intended for use in special patient populations. Here, we verified the proposed approach in a population of 35 patients with IBP signals as the reference, which guarantees statistical reliability according to the AAMI standard. Additionally, we also analyzed the performance of the proposed method with PPG features only. ME ± STD values for the SBP and DBP estimation using only PPG features were 0.11 ± 8.17 and −0.07 ± 4.14 mmHg, respectively (Supplementary Table S2).

**TABLE 3:** Performance evaluation according to the AAMI and BHS standards.

		AAMI Standard					BHS Standard		
		ME (mmHg)	STD (mmHg)	Special populations			CP at 5 mmHg	CP at 10 mmHg	CP at 15 mmHg
AAMI	SBP	5	8	≥35	BHS	Grade A	60%	85%	95%
	DBP	5	8	≥35		Grade B	50%	75%	90%
						Grade C	40%	65%	85%
Our results	SBP	−0.04	6.11	35	Our Results		67.61%	91.13%	97.33%
	DBP	0.11	3.62	35			87.04%	95.58%	99.64%

*AAMI, advancement of medical instrumentation; BHS, British hypertension society; CP, cumulative percentage; DBP, diastolic blood pressure; ME, mean error; SBP, systolic blood pressure; STD, standard deviation of error.*

**FIGURE 5:**
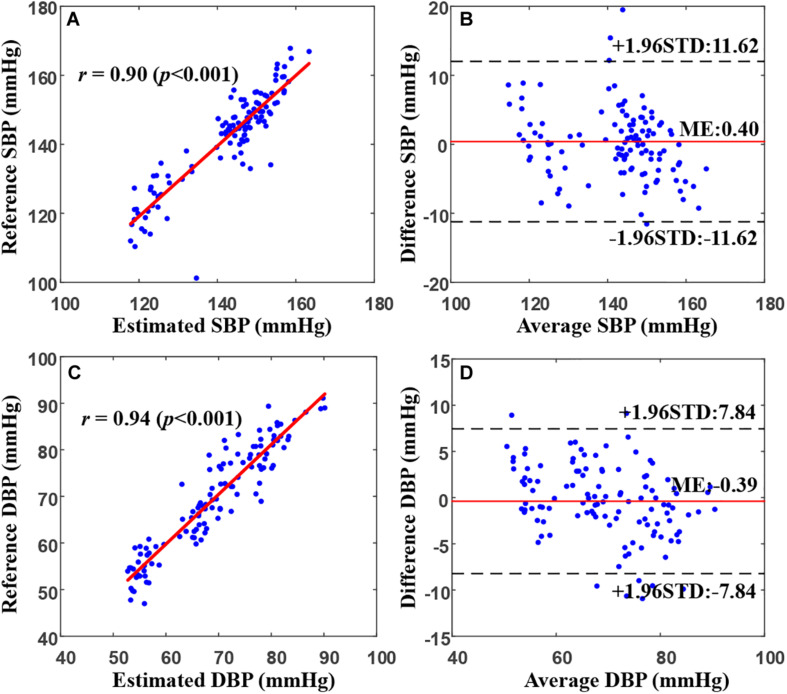
Correlation and Bland–Altman plots for estimated SBP **(A,B)** and DBP **(C,D)** values from the proposed model (by using the random forest regression model) versus the references in a representative patient. In **(B)** and **(D)**, the black dotted and solid red lines represent the ME ± 1.96 × STD. DBP, diastolic blood pressure; ME, mean error; STD, standard deviation of error; SBP, systolic blood pressure.

Figure 5 presents an example of the correlation and Bland–Altman plots for the SBP and DBP estimated by the proposed model compared with the reference BP values in a representative patient. The correlation coefficients between the estimated SBP and DBP values and the reference values were 0.90 and 0.94, respectively, indicating a very high consistency between the BP estimates and the references. The Bland–Altman plot indicated that the estimated BP values approximated the reference values very well, with >95% of the points lying within the limit of agreement in SBP and DBP estimates. In particular, for this patient, the bias of differences between our estimation and the reference values for SBP and DBP were 0.4 ± 5.93 and −0.39 ± 3.99 mmHg, respectively.

### Feature Importance Assessment

Table 4 presents the average group importance levels of the extracted features, sorted by the importance level for predicting BP. In general, the features (SYSAREA, FAS, and AS) extracted from the ascending edge of PPG were more significant in the SBP estimation than were those (DIAAREA, FDS, and DS) extracted from the descending edge of PPG. Conflicting results were observed in DBP estimation: the features (DT and DIAAREA) extracted from the descending edge of PPG played a more significant role in the DBP estimation than those (AT and SYSAREA) extracted from the ascending edge of PPG. For SBP estimation, the most critical indicator was PTTrm, followed by SYSAREA (ascending area). Correspondingly, for DBP estimation, the most important predictor was descending time (DT), followed by DIAAREA (descending area).

**TABLE 4:** Average group importance levels of PTTs and PPG features for blood pressure estimation.

Feature	SBP	Feature	DBP
PTTrm	0.1670	DT	0.2190
SYSAREA	0.1281	DIAAREA	0.1392
PTTrf	0.0889	HR	0.0886
FAS	0.0841	K	0.0884
AS	0.0778	FAS	0.0621
DT	0.0738	DS	0.0548
PIR	0.0637	AS	0.0542
DIAAREA	0.0579	PIR	0.0488
PW	0.0574	SYSAREA	0.0474
K	0.0419	FDS	0.0371
HR	0.0390	PTTrp	0.0367
AT	0.0357	PTTrf	0.0362
DS	0.0315	PW	0.0357
PTTrp	0.0278	AT	0.0267
FDS	0.0253	PTTrm	0.0250

*Abbreviations and detailed definitions shown in Table 1.*

## Discussion

In this study, different measures of PTT and several informative PPG features were calculated from ECG and PPG signals and then used to construct the BP estimation model based on various machine learning algorithms. The experimental results revealed that the combination of PTTs and PPG features in the proposed model based on machine learning algorithms could realize BP estimation with high accuracy during arrhythmias. The inherent strength of this paper is the verification of the feasibility of using ECG and PPG signals to estimate BP in patients with arrhythmias.

### Comparison With Other Works

We compared the proposed method with other works (including multi-parameter-based methods and PTT-based methods in terms of dataset type, reference BP type, and the performance of each on its own dataset). Table 5 present the results of the comparison. Although the accuracy of the proposed method was lower than that of methods in studies in healthy populations using Finapres as the reference (Ding X.-R. et al., 2016; Yan et al., 2019), it substantially outperformed methods applied in intensive care unit patients (Kachuee et al., 2017). Furthermore, compared to other studies in hypertensive and aged population using the cuff-based method as the reference (Chen et al., 2018; Miao et al., 2020), our method was verified in patients with arrhythmias using the IBP as the standard and achieved relatively good performance, suggesting the higher reliability of our proposed approach.

**TABLE 5:** Comparison with other works.

Work	Dataset	Reference BP	SBP (mmHg)			DBP (mmHg)		
			ME	STD	MAE	ME	STD	MAE
PTT and PIR (Ding X.-R. et al., 2016)	27 Healthy subjects	Finapres	−0.37	5.21	4.09	−0.18	4.13	3.18
Multi-parameter (Yan et al., 2019)	70 Healthy subjects	Finapres	0.04	5.00	–	0.01	3.69	–
PTT and heart-rate variability (Chen et al., 2018)	29 Hypertensive subjects	Auscultatory technique	0.73	10.04	–	0.90	7.10	–
Multi-parameter (Miao et al., 2020)	85 Aged subjects	Oscillometric technique	1.62	7.76	6.13	1.59	5.52	4.54
Multi-parameter (Kachuee et al., 2017)	57 ICU patients	Intra-arterial	–	–	8.21	–	–	4.31
This work	35 Arrhythmias patients	Intra-arterial	−0.04	6.11	5.89	0.11	3.62	2.59

*Abbreviations shown in Table 3.*

### Correlation Between PTT and BP During Arrhythmias

Pulse transit time is highly correlated with BP variation in healthy populations (Huynh et al., 2018; Yang and Tavassolian, 2018). However, the correlation is weak in patients with cardiovascular diseases (Wagner et al., 2010; Liu et al., 2014; Ding X. et al., 2016). For instance, Wagner et al. (2010) investigated the relationship between PTT and BP in patients with chronic heart failure, and their results revealed that the correlation coefficient between PTT and BP in patients (*r* = 0.23) was lower than that in healthy individuals (*r* = 0.73), suggesting that the relationship between PTT and BP is weak. A potential reason is that the gold reference in the previous studies was intermittent BP based on the oscillometric technique or Finapres BP, which are not very reliable methods for patients with cardiovascular diseases. Furthermore, studies using IBP as the reference indicated that the beat-to-beat PTT correlated well with IBP (*r* = 0.81) in hypertension patients (Kim et al., 2013), particularly when BP has a wide variation in intensive care unit patients (Escobar-Restrepo et al., 2018). Because IBP is recommended for patients with arrhythmias, whether the IBP–PTT association mentioned above sustains during arrhythmias should be investigated to provide accurate cuff-less continuous BP measurement in arrhythmia patients.

**FIGURE 6:**
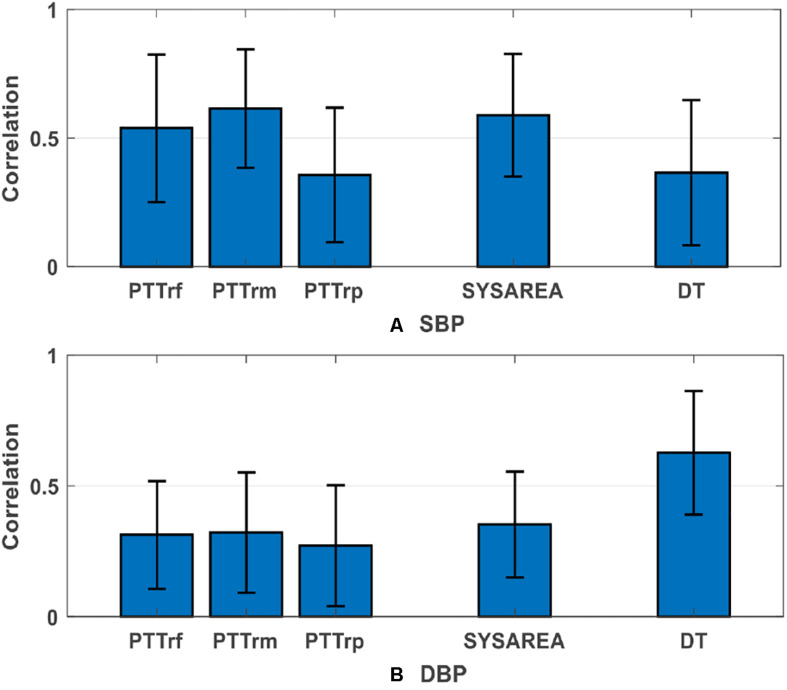
Group average absolute correlations (mean value ± standard deviation) between three PTTs and the PPG features that most relevant to blood pressure versus **(A)** SBP and **(B)** DBP. DBP, diastolic blood pressure; SBP, systolic blood pressure; other abbreviations and detailed definitions show in Table 1.

In this study, we evaluated three measures of PTT (Features 1 to 3, listed in Table 1), namely PTTrf, PTTrm, and PTTrp, for BP estimation during arrhythmias, with IBP signals as the reference. Our analysis results indicated that PTT features play a more important role in SBP estimation than PPG features. Supplementary Table S2 presents the overall performance comparison between the proposed method with and without PTT features (PTTrf, PTTrm, and PTTrp) for BP estimation. After removing the PTT features, the performance of the proposed method decreased, especially the SBP estimation did not meet the AAMI standard. Therefore, we recommend using the combination of PTT and PPG features to obtain a more accurate BP estimation under arrhythmias. Moreover, we found that PTTrm was more critical for SBP estimation than other PTTrf and PTTrp. This result is similar to that of our previous study (Miao et al., 2017), in which the PTT_MaxDeri that was calculated from the peak of the first derivation of PPG demonstrated a more significant role than those derived from the foot and peak of PPG in the SBP estimation. Moreover, PTTrm was well correlated with SBP (*r* = 0.61 ± 0.23; Figure 6) during arrhythmias. These findings provide a potential insight to estimate continuous BP from ECG and PPG signals in patients with arrhythmias. The physiological significance of the correlation between PTT (PTTrm) and BP can be reasonably deduced. Unstable cardiac contraction and insufficient effective cardiac output induced by reduced ventricular filling time are observed during arrhythmias (e.g., supraventricular arrhythmias) (Hebbar and Hueston, 2002). In terms of physiology, under the same conditions for peripheral resistance and arterial stiffness, the attenuated cardiac contraction during arrhythmias can result in low BP. Besides, studies (Eliakim et al., 1971; Weinman et al., 1971) on the changes in pulse wave velocity (the reciprocal of PTT) in patients with arrhythmias have demonstrated that PTT calculated from ECG and PPG is inversely related to the duration of the preceding cardiac cycles during arrhythmias, particularly in shorter cycles. That is, when the heartbeat interval is shortened during arrhythmias, PTT increases. Therefore, PTT is correlated with beat-to-beat BP during arrhythmias.

### PPG Features for BP Estimation

Photoplethysmogram is a direct reflection of blood volume changes in the microvascular bed of tissue and then in the artery; thus, it correlates with BP variation. Furthermore, because of the changes in blood perfusion in peripheral blood vessels, PPG reflects not only the blood ejection from the heart but also the condition of peripheral arteries closely related to BP.

In previous studies, several parameters have been extracted from PPG signals and considered along with PTT for BP estimation. For instance, Lin et al. (2018) and Baek et al. (2009) have indicated that useful features obtained from the second derivative wave of PPG have the potential to be non-invasive surrogate markers for BP estimation. However, all the studies mentioned have mainly focused on a healthy population. The extracted features they proposed lacks accuracy in patients because of the inappropriate PPG signal. In this study, we extracted several crucial features that could be accurately calculated from the PPG signal to estimate the BP. Feature importance assessment results demonstrated that among all the PPG features, systolic area (SYSAREA), mainly extracted from the systole (ascending edge) of PPG signals, is the most critical feature for SBP estimation (importance value = 0.1281), whereas DT, extracted from the diastole (descending edge) of PPG signals, is the most important PPG feature (importance value = 0.2190). Figure 6 presents the average group absolute correlation coefficients between the reference BP versus the corresponding extracted features, which are also consistent with the findings above. These findings are reasonable because SBP variation mainly depends on cardiac contraction and relevant stroke volume reflected in the systole of PPG signal, whereas DBP variation mostly depends on peripheral resistance and arterial stiffness reflected in the diastole of the PPG signal.

### Clinical Application Prospect

Experimental results based on 35 clinically stable patients indicated that beat-to-beat BP can be accurately estimated from ECG and PPG signals under arrhythmias via machine learning algorithms. Due to the large individual differences in BP changes under arrhythmias, personalized modeling was used in this study. However, there are still potential clinical application prospects of this work in the field of continuous BP measurement. In the clinical setting, the intra-arterial method is recommended for the BP measurement of patients with arrhythmias (Alpert et al., 2006). However, prolonged insertion of a catheter into the artery can cause risks such as bleeding, thrombosis, arterial damage for patients (Bedford, 1978; Scheer et al., 2002). Adopting a fusion strategy, in which individual initial calibration is first performed through the IBP measured by artery intubation, followed by long-term tracking using the ECG-PPG–based method, would reduce the risks associated with prolonged artery cannulation and provide a non-invasive and accurate long-term continuous BP monitoring for patients with arrhythmias. This strategy is similar to the artery applanation tonometry and volume clamp techniques, which also require the cuff-based BP for initial calibration (Chung et al., 2013). Moreover, this work provides the experimental fundament for establishing a universal BP estimation model under arrhythmias in the future.

### Strengths and Limitations

Our study has several strengths. Firstly, this study was the first to investigate the potential of using ECG and PPG signals for cuff-less and continuous BP estimation under arrhythmias. Secondly, an invasive method, which directly measures the BP inside the vessels, was used as the gold reference to evaluate the performance of the proposed method. Finally, we have identified crucial features for BP estimation under arrhythmias from ECG and PPG signals. However, there are some limitations to the present study. Firstly, IBP waveforms were measured at the right femoral artery to prevent additional injury during the operation, whereas the PPG sensor was placed on the left index finger. The inconsistency between BPs in the right artery and left artery may have also led to a certain degree of error in the model. Secondly, the practical meaning of PTTrm and DT, which are of most importance for SBP and DBP estimation respectively, are still unclear and need to be further studied. Thirdly, as different patients may have different hemodynamic responses to BP changes, personalized modeling was used in this study. Although personalized modeling has its shortcomings, it has been widely adopted in previous studies to reduce individual differences (Ding X.-R. et al., 2016; Miao et al., 2017, 2020). Finally, due to the complexity in collecting IBP for a long time, only 35 clinically stable patients were enrolled in our study for short-term BP estimation. In the future, a larger cohort study, including more patients with arrhythmias, will be conducted to discover short- and long-term indicators and then develop a universal BP estimation model under arrhythmias.

## Conclusion

In this study, for the first time, we investigated the potential of a cuff-less and continuous BP estimation approach from ECG and PPG signals during arrhythmias. Thirty-five clinically stable patients with arrhythmias who underwent radiofrequency ablation were enrolled for simultaneously ECG, PPG, and IBP signal collection. Fifteen features were extracted from ECG and PPG and then used to construct the BP model with IBP as the gold reference based on machine learning algorithms. The proposed approach complied with the AAMI and Grade A of BHS standards in SBP and DBP estimation during arrhythmias, indicating that the feasibility of using ECG and PPG signals to estimate continuous BP in an indirect way for patients with arrhythmias. Further research is needed in the future to deeply explore the value of ECG and PPG signals in estimating continuous BP in more patients with arrhythmias, to promote the clinical application prospect.

## Data Availability Statement

The raw data supporting the conclusions of this article will be made available by the authors, without undue reservation.

## Ethics Statement

The studies involving human participants were reviewed and approved by institutional ethics review board of Fuwai Hospital, Chinese Academy of Medical Sciences (Approval No. 2019-1239). The protocol was registered on www.chictr.org.cn (registration number: ChiCTR2000031170). The patients/participants provided their written informed consent to participate in this study.

## Author Contributions

FM, YL, and MT conceived and designed research. MT and BZ conducted experiments. ZL and BZ analyzed data and wrote the manuscript. FM and YL revised the manuscript. All authors read and approved the final manuscript.

## Conflict of Interest

The authors declare that the research was conducted in the absence of any commercial or financial relationships that could be construed as a potential conflict of interest.

## References

[B1] Alpert B. Mccrindle B. Daniels S. Dennison B. Hayman L. Jacobson M. (2006). Recommendations for blood pressure measurement in human and experimental animals; part 1: blood pressure measurement in humans. *Hypertension* 48:e3. 10.1161/01.HYP.0000229661.06235.08 16769991

[B2] Arza A. Lázaro J. Gil E. Laguna P. Bailón R. (2013). “Pulse transit time and pulse width as potential measure for estimating beat-to-beat systolic and diastolic blood pressure,” in *Proceedings of the Computing in Cardiology*, Zaragoza.

[B3] Association for the Advancement of Medical Instrumentation [AAMI] (2009). *Non-Invasive Sphygmomanometers - Part 2: Clinical Validation of Automated Measurement Type.* Arlington, VA: Association for the Advancement of Medical Instrumentation.

[B4] Awad M. Khanna R. (2015). “Support vector regression,” in *Efficient Learning Machines: Theories, Concepts, and Applications for Engineers and System Designers*, eds Awad M. Khanna R. (Berkeley, CA: Apress), 67–80.

[B5] Baek H. J. Kim K. K. Kim J. S. Lee B. Park K. S. (2009). Enhancing the estimation of blood pressure using pulse arrival time and two confounding factors. *Physiol. Meas.* 31:145 10.1088/0967-3334/31/2/002 20009186

[B6] Bedford R. F. (1978). Long-term radial artery cannulation: effects on subsequent vessel function. *Crit. Care Med.* 6 64–67. 10.1097/00003246-197801000-00016 639534

[B7] Breiman L. (2001). Random forests. *Mach. Learn.* 45 5–32.

[B8] Chen Y. Shi S. Liu Y.-K. Huang S.-L. Ma T. (2018). Cuffless blood-pressure estimation method using a heart-rate variability-derived parameter. *Physiol. Meas.* 39:095002. 10.1088/1361-6579/aad902 30089101

[B9] Chung E. Chen G. Alexander B. Cannesson M. (2013). Non-invasive continuous blood pressure monitoring: a review of current applications. *Front. Med.* 7:91–101. 10.1007/s11684-013-0239-5 23345112

[B10] Cohen D. L. Townsend R. R. (2017). Blood Pressure in Patients With Atrial Fibrillation: Part 1—-Measurement. *J. Clin. Hypertens.* 19 98–99. 10.1111/jch.12905 27543382 PMC8031305

[B11] Cutler A. Cutler D. R. Stevens J. R. (2012). “Random Forests,” in *Ensemble Machine Learning: Methods and Applications*, eds Zhang C. Ma Y. (Boston, MA: Springer US), 157–175.

[B12] Ding X. Zhang Y.-T. (2019). Pulse transit time technique for cuffless unobtrusive blood pressure measurement: from theory to algorithm. *Biomed. Eng. Lett.* 9 37–52. 10.1007/s13534-019-00096-x 30956879 PMC6431352

[B13] Ding X.-R. Zhang Y.-T. Liu J. Dai W.-X. Tsang H. K. (2016). Continuous cuffless blood pressure estimation using pulse transit time and photoplethysmogram intensity ratio. *IEEE Trans. Biomed. Eng.* 63 964–972. 10.1109/tbme.2015.2480679 26415147

[B14] Ding X. Zhang Y. Tsang H. K. (2016). Impact of heart disease and calibration interval on accuracy of pulse transit time-based blood pressure estimation. *Physiol. Meas.* 37 227–237. 10.1088/0967-3334/37/2/227 26767518

[B15] Eliakim M. Sapoznikov D. Weinman J. (1971). Pulse wave velocity in healthy subjects and in patients with various disease states. *Am. Heart. J.* 82 448–457. 10.1016/0002-8703(71)90229-8 5111225

[B16] Escobar-Restrepo B. Torres-Villa R. Kyriacou P. A. (2018). Evaluation of the linear relationship between pulse arrival time and blood pressure in ICU patients: potential and limitations. *Front. Physiol.* 9:1848. 10.3389/fphys.2018.01848 30622482 PMC6308183

[B17] Freund Y. Schapire R. Abe N. (1999). A short introduction to boosting. *J. Jpn. Soc. Artif. Intellig.* 14:1612.

[B18] Go A. S. Hlatky M. A. Liu T. I. Fan D. Garcia E. A. Sung S. H. (2018). Contemporary burden and correlates of symptomatic paroxysmal supraventricular tachycardia. *J. Am. Heart Assoc.* 7:e008759. 10.1161/JAHA.118.008759 PMC6064827 29982228

[B19] Hatch F. Lancaster M. K. Jones S. A. (2011). Aging is a primary risk factor for cardiac arrhythmias: disruption of intracellular Ca2+ regulation as a key suspect. *Expert Rev. Cardiovasc. Ther.* 9 1059–1067. 10.1586/erc.11.112 21878050

[B20] Hebbar K. A. Hueston W. J. (2002). Management of common arrhythmias: Part I. *Supraventricular arrhythmias*. *Am. Fam. Phys.* 65:2479. 12086237

[B21] Huynh T. H. Jafari R. Chung W.-Y. (2018). Noninvasive cuffless blood pressure estimation using pulse transit time and impedance plethysmography. *IEEE Trans. Biomed. Eng.* 66 967–976. 10.1109/tbme.2018.2865751 30130167

[B22] Ilies C. Grudev G. Hedderich J. Renner J. Steinfath M. Bein B. (2015). Comparison of a continuous noninvasive arterial pressure device with invasive measurements in cardiovascular postsurgical intensive care patients: A prospective observational study. *Eur. J. Anaesthesiol.* 32:20. 10.1097/eja.0000000000000136 25105850

[B23] Jenkins J. M. Wu D. Arzbaecher R. (1979). Computer diagnosis of supraventricular and ventricular arrhythmias. A new esophageal technique. *Circulation* 60 977–987. 10.1161/01.cir.60.5.977 487556

[B24] Kachuee M. Kiani M. M. Mohammadzade H. Shabany M. (2017). Cuffless blood pressure estimation algorithms for continuous health-care monitoring. *IEEE Trans. Biomed. Eng.* 64 859–869. 10.1109/tbme.2016.2580904 27323356

[B25] Khurshid S. Choi S. H. Weng L.-C. Wang E. Y. Trinquart L. Benjamin E. J. (2018). Frequency of cardiac rhythm abnormalities in a half million adults. *Circulation* 11:e006273. 10.1161/CIRCEP.118.006273 PMC6051725 29954742

[B26] Kim S. H. Lilot M. Sidhu K. S. Rinehart J. Cannesson M. (2014). Accuracy and precision of continuous noninvasive arterial pressure monitoring compared with invasive arterial pressure a systematic review and meta-analysis. *Anesthesiology* 120 1080–1097. 10.1097/aln.0000000000000226 24637618

[B27] Kim S.-H. Song J.-G. Park J.-H. Kim J.-W. Park Y.-S. Hwang G.-S. (2013). Beat-to-beat tracking of systolic blood pressure using noninvasive pulse transit time during anesthesia induction in hypertensive patients. *Anesthesia Anal.* 116 94–100. 10.1213/ane.0b013e318270a6d9 23223109

[B28] Kligfield P. Gettes L. S. Bailey J. J. Childers R. Deal B. J. Hancock E. W. (2007). Recommendations for the Standardization and Interpretation of the Electrocardiogram. Part I: The Electrocardiogram and Its Technology. A Scientific Statement From the American Heart Association Electrocardiography and Arrhythmias Committee, Council on Clin. *Circulation* 4:394. 10.1016/j.hrthm.2007.01.027 17341413

[B29] Lee J.-H. Lin C.-J. (2000). *Automatic Model Selection for Support Vector Machines. Technical Report, Department of Computer Science and Information Engineering, National Taiwan University*. Available online at: http://citeseerx.ist.psu.edu/viewdoc/download?doi=10.1.1.32.5059&rep=rep1&type=pdf

[B30] Liaw A. Wiener M. (2002). Classification and regression by randomForest. *R News* 2 18–22.

[B31] Lin W.-H. Ji N. Wang L. Li G. (2019). “A characteristic filtering method for pulse wave signal quality assessment,” in *Proceedings of the 2019 41st Annual International Conference of the IEEE Engineering in Medicine and Biology Society (EMBC)*, (Piscataway: IEEE), 603–606. 10.1109/EMBC.2019.8856811 31945970

[B32] Lin W.-H. Wang H. Samuel O. W. Liu G. Huang Z. Li G. (2018). New photoplethysmogram indicators for improving cuffless and continuous blood pressure estimation accuracy. *Physiol. Meas.* 39:025005. 10.1088/1361-6579/aaa454 29319536

[B33] Liu J. Yan B. P. Zhang Y.-T. Ding X.-R. Su P. Zhao N. (2018). Multi-wavelength photoplethysmography enabling continuous blood pressure measurement with compact wearable electronics. *IEEE Trans. Biomed. Eng.* 66 1514–1525. 10.1109/tbme.2018.2874957 30307851

[B34] Liu Z.-D. Liu J.-K. Wen B. He Q.-Y. Li Y. Miao F. (2018). Cuffless blood pressure estimation using pressure pulse wave signals. *Sensors* 18:4227. 10.3390/s18124227 30513838 PMC6308537

[B35] Liu Q. Yan B. P. Yu C. M. Zhang Y. T. Poon C. C. (2014). Attenuation of systolic blood pressure and pulse transit time hysteresis during exercise and recovery in cardiovascular patients. *IEEE Trans. Biomed. Eng.* 61 346–352. 10.1109/tbme.2013.2286998 24158470

[B36] Miao F. Fu N. Zhang Y.-T. Ding X.-R. Hong X. He Q. (2017). A novel continuous blood pressure estimation approach based on data mining techniques. *IEEE J. Biomed. Health Inform.* 21 1730–1740. 10.1109/jbhi.2017.2691715 28463207

[B37] Miao F. Liu Z. Liu J. Wen B. Li Y. (2020). Multi-sensor Fusion Approach for Cuff-less Blood Pressure Measurement. *IEEE J. Biomed. Health Inform.* 24 79–91. 10.1109/jbhi.2019.2901724 30892255

[B38] Mukkamala R. Hahn J.-O. Inan O. T. Mestha L. K. Kim C.-S. Toreyin H. (2015). Toward ubiquitous blood pressure monitoring via pulse transit time: theory and practice. *IEEE Trans. Biomed. Eng.* 62 1879–1901. 10.1109/tbme.2015.2441951 26057530 PMC4515215

[B39] O’Brien E. Petrie J. Littler W. Swiet M. De Padfield P. L. O’malley K. (1990). The British Hypertension Society protocol for the evaluation of automated and semi-automated blood pressure measuring devices with special reference to ambulatory systems. *J. Hypertens.* 8 607–619. 10.1097/00004872-199007000-00004 2168451

[B40] Pedregosa F. Varoquaux G. Gramfort A. Michel V. Thirion B. Grisel O. (2011). Scikit-learn: Machine learning in Python. *J. Mach. Learn. Res.* 12 2825–2830.

[B41] Peter L. Noury N. Cerny M. (2014). A review of methods for non-invasive and continuous blood pressure monitoring: Pulse transit time method is promising? *IRBM* 35 271–282. 10.1016/j.irbm.2014.07.002

[B42] Quinlan J. R. (1986). Induction of decision trees. *Mach. Learn.* 1 81–106.

[B43] Scheer B. V. Perel A. Pfeiffer U. J. (2002). Clinical review: complications and risk factors of peripheral arterial catheters used for haemodynamic monitoring in anaesthesia and intensive care medicine. *Crit. Care* 6:199. 10.1186/cc1489 PMC137445 12133178

[B44] Somayyeh M. S. Mohammad F. Mostafa C. Mohammad H. Maryam M. Yadollah G. (2019). Blood pressure estimation from appropriate and inappropriate PPG signals using A whole-based method. *Biomed. Signal Process. Control* 47 196–206. 10.1016/j.bspc.2018.08.022

[B45] Tse H.-F. Wang Y.-J. Ai-Abdullah M. A. Pizarro-Borromeo A. B. Chiang C.-E. Krittayaphong R. (2013). Stroke prevention in atrial fibrillation—an Asian stroke perspective. *Heart Rhythm* 10 1082–1088. 23501173 10.1016/j.hrthm.2013.03.017

[B46] Wagner D. R. Roesch N. Harpes P. Körtke H. Plumer P. Saberin A. (2010). Relationship between pulse transit time and blood pressure is impaired in patients with chronic heart failure. *Clin. Res. Cardiol.* 99 657–664. 10.1007/s00392-010-0168-0 20473677

[B47] Weinman J. Sapoznikov D. Eliakim M. (1971). Arterial pulse wave velocity and left ventricular tension period in cardiac arrhythmias. *Cardiovasc. Res.* 5 513–523. 10.1093/cvr/5.4.513 4110609

[B48] Yan W. Peng R. Zhang Y. Ho D. (2019). Cuffless continuous blood pressure estimation from pulse morphology of photoplethysmograms. *IEEE Access.* 7 141970–141977. 10.1109/access.2019.2942936

[B49] Yang C. Tavassolian N. (2018). Pulse transit time measurement using seismocardiogram, photoplethysmogram, and acoustic recordings: evaluation and comparison. *IEEE J. Biomed. Health Inform.* 22 733–740. 10.1109/jbhi.2017.2696703 28436909

[B50] Yildirir A. Batur M. Oto A. (2002). Hypertension and arrhythmia: blood pressure control and beyond. *Europace* 4 175–182. 10.1053/eupc.2002.0227 12135251

