# Supplementary material for: Continuous Blood Pressure Estimation From Electrocardiogram and Photoplethysmogram During Arrhythmias
Source: Front Physiol. 2020 Sep 9;11:575407. doi: 10.3389/fphys.2020.575407 (PMC7509183; doi:10.3389/fphys.2020.575407)
Supplement: Supplementary file 1 [file Table_1.DOCX]

Supplementary Material

# Supplementary Text S1

Lets $\{x_{1},x_{2},\cdots x_{n}\}$ be the reference BP values from the IBP signals and $\{y_{1},y_{2},\cdots y_{n}\}$ the estimated BP values with the proposed model. Root-mean-square error (RMSE), mean error (ME), standard deviation of error (STD), mean absolute error (MAE) and correlation coefficient (CC) between the predicated BP values with the proposed model and the reference BP can be calculated as follows:

$$\begin{aligned} RMSE=\sqrt{\frac{\sum_{i=1}^{n} \left( y_{i}-x_{i} \right)^{2}}{n}}\#\left( 1 \right) \end{aligned}$$

$$\begin{aligned} ME=\frac{\sum_{i=1}^{n} \left( y_{i}-x_{i} \right)}{n}\#\left( 2 \right) \end{aligned}$$

$$\begin{aligned} STD=\sqrt{\frac{\sum_{i=1}^{n} \left( y_{i}-x_{i}-ME \right)^{2}}{n-1}}\#\left( 3 \right) \end{aligned}$$

$$\begin{aligned} MAE=\frac{\sum_{i=1}^{n} \left| y_{i}-x_{i} \right|}{n}\#\left( 4 \right) \end{aligned}$$

$$\begin{aligned} CC=\frac{\sum_{i=1}^{n} \left( x_{i}-\bar{x} \right)\left( y_{i}-\bar{y} \right)}{\sqrt{\sum_{i=1}^{n} \left( x_{i}-\bar{x} \right)^{2}}\sqrt{\sum_{i=1}^{n} \left( y_{i}-\bar{y} \right)^{2}}}\#\left( 5 \right) \end{aligned}$$

Where $\bar{x}$ and $\bar{y}$ are the average of $\{x_{1},x_{2},\cdots x_{n}\}$ and $\{y_{1},y_{2},\cdots y_{n}\}$, respectively.

# Supplementary Tables

**Table S1.** Performance of the proposed method for the BP estimation under different types of beats

| Type of beat | Number | SBP Estimation | |  | DBP Estimation | |
| --- | --- | --- | --- | --- | --- | --- |
|  |  | ME (mmHg) | STD (mmHg) |  | ME (mmHg) | STD (mmHg) |
| Sinus rhythm | 1 223 | -0.11 | 5.13 |  | 0.14 | 3.38 |
| Ventricular arrhythmias | 694 | 0.42 | 7.69 |  | 0.36 | 3.98 |
| Supraventricular arrhythmias | 1 654 | -0.18 | 6.05 |  | -0.03 | 3.64 |
| **Overall** | **3 571** | **-0.04** | **6.11** |  | **0.11** | **3.62** |

BP: blood pressure; SBP: systolic blood pressure; DBP: diastolic blood pressure; ME: mean error (ME); STD: standard deviation of error

**Table S2**. Performance comparison between the proposed method and without PTT features

|  | SBP Estimation | |  | DBP Estimation | |
| --- | --- | --- | --- | --- | --- |
|  | ME (mmHg) | STD (mmHg) |  | ME (mmHg) | STD (mmHg) |
| AAMI Standard | ≤ 5 | ≤ 8 |  | ≤ 5 | ≤ 8 |
| Proposed method with PPG and PTT features | -0.04 | 6.11 |  | 0.11 | 3.62 |
| Proposed method with PPG features only | 0.11 | 8.17 |  | -0.07 | 4.14 |

AAMI: Advancement of Medical Instrumentation; SBP: systolic blood pressure; DBP: diastolic blood pressure; ME: mean error; STD: standard deviation of error; PTT: pulse transit time; PPG: photoplethysmogram

# Supplementary Figures


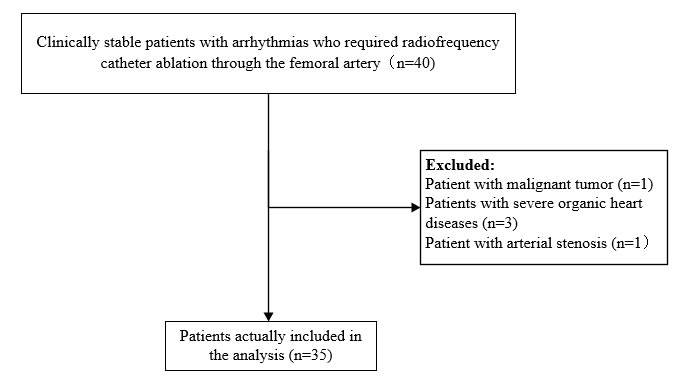


**Supplementary Figure S1.** Flowchart of the study population.
